# Single-Layer ZnO Hollow Hemispheres Enable High-Performance Self-Powered Perovskite Photodetector for Optical Communication

**DOI:** 10.1007/s40820-021-00596-5

**Published:** 2021-02-12

**Authors:** Xiyan Pan, Jianqiang Zhang, Hai Zhou, Ronghuan Liu, Dingjun Wu, Rui Wang, Liangping Shen, Li Tao, Jun Zhang, Hao Wang

**Affiliations:** Hubei Key Laboratory of Ferro and Piezoelectric Materials and Devices, School of Microelectronics and Faculty of Physics and Electronic Science, Hubei University, Wuhan, 430062 People’s Republic of China

**Keywords:** Hemisphere array, Perovskite, Photodetector, Reflection reduction, Optical communication

## Abstract

The carrier transport layer with reflection reduction morphology has attracted extensive attention for improving the utilization of light. Herein, we introduced single-layer hollow ZnO hemisphere arrays (ZHAs) behaving light trapping effect as the electron transport layer in perovskite photodetectors (PDs). The single-layer hollow ZHAs can not only reduce the reflection, but also widen the angle of the effective incident light and especially transfer the distribution of the optical field from the ZnO/FTO interface to the perovskite active layer confirmed by the 3D finite-difference time-domain simulation. These merits benefit for the generation, transport and separation of carriers, improving the light utilization efficiency. Finally, our optimized FTO/ZHA/CsPbBr_3_/carbon structure PDs showed high self-powered performance with a linear dynamic range of 120.3 dB, a detectivity of 4.2 × 10^12^ Jones, rise/fall time of 13/28 µs and the *f*_−3 dB_ of up to 28 kHz. Benefiting from the high device performance, the PD was demonstrated to the application in the directional transmission of encrypted files as the signal receiving port with super high accuracy. This work uniquely utilizes the features of high-performance self-powered perovskite PDs in optical communication, paving the path to wide applications of all-inorganic perovskite PDs.

**Supplementary Information:**

The online version of this article (10.1007/s40820-021-00596-5) contains supplementary material, which is available to authorized users.

## Highlights

- Single-layer hollow ZnO hemispherical arrays behaving light trapping effect as the electron transport layer in perovskite photodetectors were first introduced.
- Our photodetectors showed high self-powered performances with a LDR of 120.3 dB, a detectivity of 4.2 × 10^12^ Jones, rise/fall time of 13/28 µs and the *f*_−3 dB_ of up to 28 kHz.
- Benefiting from the high device performance, the photodetector was demonstrated to the directional transmission of encrypted files as the signal receiving port with super high accuracy.

**Supplementary Information:**

The online version of this article (10.1007/s40820-021-00596-5) contains supplementary material, which is available to authorized users.

## Graphical abstract

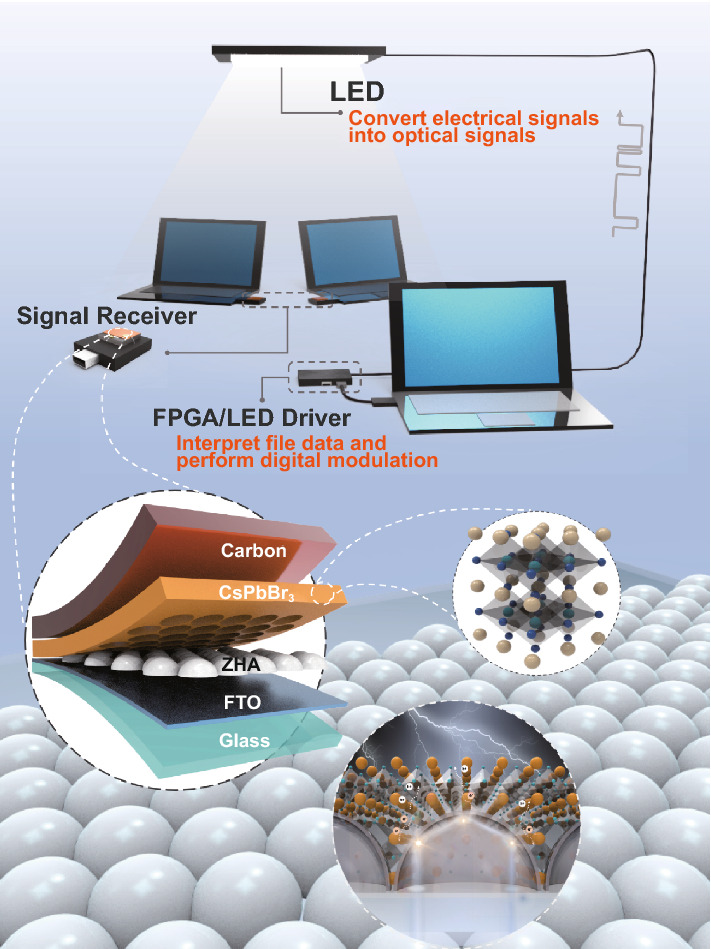

## Introduction

The advantages of the organic–inorganic hybrid halide perovskite, such as adjustable band gap, high optical absorption coefficient, long carrier diffusion length and carrier lifetime, high carrier mobility and low trap state density, enable the efficiency of the perovskite solar cells (SCs) climbing from 3.8% to 25.2% in the past decade [1–15]. However, due to the instability of organic cations, the commercialization of organic–inorganic halide perovskite optoelectronic devices is limited. Compared with organic halide perovskite, inorganic halide perovskite (CsPbX_3_, X = I, Br, Cl) has attracted more and more extensive attention on account of its better stability [16–29]. Self-powered photodetectors (PDs) based on all-inorganic halide perovskite can not only work at a low bias or even zero bias to obtain low-power devices, but also be easier to achieve higher detectivity due to the lower dark current, and these merits enable the self-powered PDs to be used in a wider field.

Conventionally, self-powered PDs are based on two kinds of structures, namely Schottky barrier type and PIN junction type (or PN junction type). Compared with the former, the latter can greatly reduce the dark current of the device, enhance carrier transmission and inhabit carrier recombination due to the existence of the carrier (electron/hole) transport layers, thereby improving the performances of the device (especially the switching ratio and detectivity) [30–36]. In addition, the carrier transport layer can effectively prevent the physical and chemical reaction between the electrode and the perovskite film, improving the device stability [37–42]. Moreover, the carrier transport layer with special morphology can effectively reduce the reflectivity of incident light and improve the utilization of light to enhance the device performances. Sargent’s team [43] found that the fully textured structure minimized reflection losses and improve the efficiency of light trapping, which was crucial to the device efficiency. Tian et al. [44] designed a nested inverse opal, which optimized the electron transport layer to enhance light capture, and the final device showed a high responsivity of 473 mA W^−1^ and the detectivity reaching 1.35 × 10^13^ Jones at 0 bias.

Herein, single-layer hollow ZnO hemisphere arrays (ZHAs) fabricated by using a polystyrene (PS) microsphere template were introduced to be the electron transport layer in perovskite PDs for enhancing light capture efficiency. In addition, compared with perovskite plane device, our ZHA-based device can enable the main light field to be distributed in the perovskite, benefitting the generation and separation of the carriers. Finally, our FTO/ZHA/CsPbBr_3_/carbon structure self-powered PDs showed high performance with a linear dynamic range (LDR) of 120.3 dB, a detectivity of 4.2 × 10^12^ Jones, rise/fall time of 13/28 µs and the *f*_−3 dB_ of up to 28 kHz. Benefiting from the high device performance, the PD was applied to the directional transmission of encrypted files, in which the signal was sent by controlling the high-speed flashing of indoor light-emitting diodes (LEDs), and the PD as the signal receiving port converted the light into electrical signals, which were identified and transcoded from the controller through the field programmable gate array (FPGA), thus realizing the directional transmission of files with super high accuracy.

## Experimental Section

### Device Fabrication

#### Single-Layer ZHA Preparation

Ten-microliter ethanol dispersion of PS microspheres with a diameter of about 1 μm was dropped into a container filled with deionized water. When the dispersion was evenly dispersed on the water surface, 1 mL of sodium dodecyl sulfate (SDS) aqueous solution (10 wt%) was dropped slowly from the edge of the container along the wall. After the SDS solution driving the PS balls on the water surface to the other end to form a dense layer, the FTO substrate was inserted obliquely into the water, moved under the dense layer of PS microspheres and lifted slowly to transfer the PS balls to the substrate. Then the glass substrate with PS balls was dried on a hot stage at 60 ℃ for 30 min, and then, single-layer PS balls were obtained. After that, the single-layer PS ball/FTO substrate was sputtered with 200 nm ZnO and then placed in an annealing furnace at 500 ℃ for 1 h to obtain single-layer hollow ZHAs.

#### CsPb_2_Br_5_ Powder Preparation

PbBr_2_ (14.62 g, 40 mmol) was dissolved in 30 mL of aqueous HBr (48%) solution and stirred to form a transparent solution. Meanwhile, CsBr (4.26 mg, 20 mmol) was dissolved in 10 mL of H_2_O. When the two kinds of the solution were mixed together, a white precipitate was formed immediately. The precipitate was filtered, then washed three times with anhydrous methanol and dried under vacuum. The CsPb_2_Br_5_ powder was obtained.

#### ZHA/CsPbBr_3_ Self-Powered PD Preparation

1 mmol of CsPb_2_Br_5_ powder was dissolved in 1 mL of dimethyl sulfoxide (DMSO) (99.9%) and stirred for 12 h as the CsPb_2_Br_5_ precursor solution. The precursor solution was spin-coated on the ZHA/FTO substrate at a speed of 3000 rpm for 30 s and then annealed at 200 °C for 20 min. After that, 10 mg mL^−1^ CsBr/methanol (99%) solution was spin-coated at a speed of 3000 rpm for 40 s and annealed at 70 °C for 1 h. Then the carbon electrode was fabricated by doctor-blading process, and the ZHA/CsPbBr_3_ self-powered PDs were prepared.

### Characterization

High-resolution scanning electron microscopy (SEM) images were recorded by field emission scanning electron microscopy (FESEM, JEOL, JSM-6700F), the X-ray diffraction (XRD) pattern was carried out by using a D8 FOCUS X-ray diffractometer, and the absorption spectra were measured by a UV–Vis spectrophotometer. The current–voltage (I–V) and current–time (*I*–*t*) curves were recorded by using a Keithley 2400 sourcemeter. An oscilloscope was used to characterize the fast response time of our devices. A 473-nm laser was used as the light source, and its optical power was calibrated by a standard Si diode. The photoluminescence (PL) spectrums and the time-resolved photoluminescence (TRPL) decay spectrums of the samples were measured by the time-correlated single-photon counting technique (FluoTime 300, Pico Quant GmbH).

### Calculation

The simulation of light field distribution was performed by the Lumerical finite-difference time-domain (FDTD) software packages. The models of ZHA/CsPbBr_3_ and planar-ZnO/CsPbBr_3_ were determined according to the geometric parameters of real devices based on the SEM images; 550 and 473 nm plane waves were chosen as the light sources, and perfectly matched layer (PML) boundary conditions were adopted.

## Results and Discussion

**Fig. 1:**
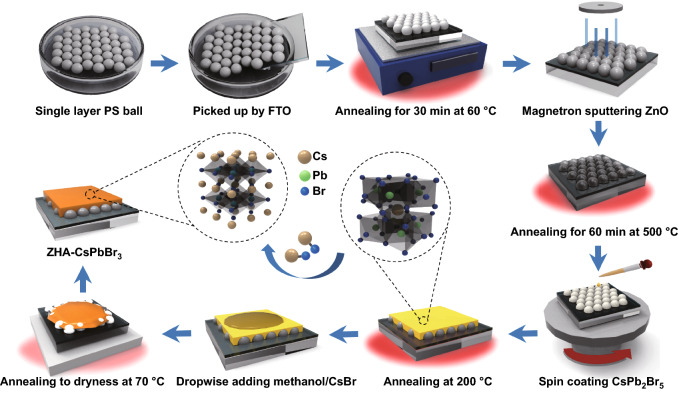
Preparation process of the ZHA/CsPbBr_3_ structure

Figure 1 shows the preparation process of the ZHA/CsPbBr_3_ structure. Firstly, 10 μL of the ethanol dispersion of PS microspheres with a diameter of about 1 μm was dripped into a container filled with deionized water. When the dispersion was evenly dispersed on the water surface, 1 mL of sodium dodecyl sulfate (SDS) aqueous solution (10 wt%) was used to drive the PS balls for obtaining a dense PS layer on the water surface. Then, the FTO substrate was used to pick up the PS microspheres and annealed at 60 °C for 30 min, and single-layer PS balls on the FTO substrate were obtained (Fig. S1a, c). After that, 200 nm ZnO was sputtered on the single-layer PS balls, following by annealing at 500 °C for 60 min to obtain a ZHA electron transport layer (Fig. S1b, d). To fabricate the ZHA/perovskite device, the CsPb_2_Br_5_ precursor solution was spin-coated on the ZHA electron transport layer due to its high solubility, and then the samples were soaked with CsBr methanol solution to obtain a high-quality CsPbBr_3_ light-absorbing layer [23]. The detailed experiment procedures can be seen in the experimental part.

**Fig. 2:**
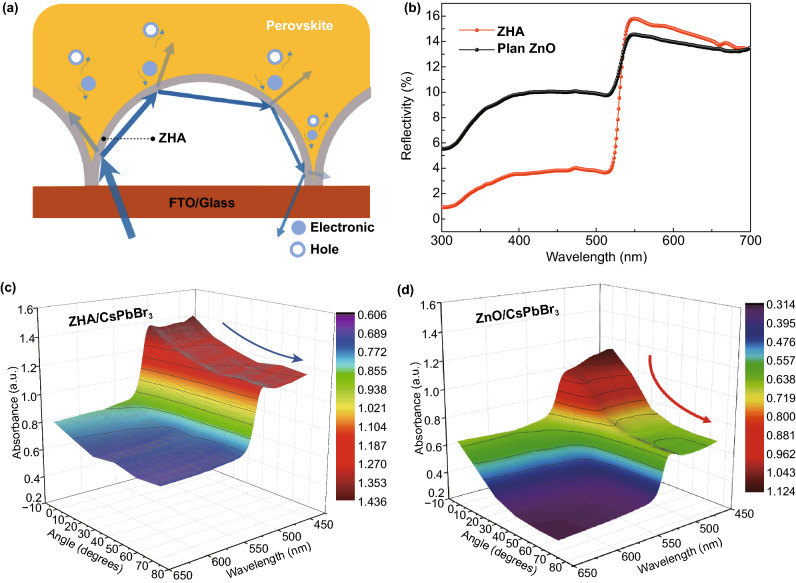
**a** Schematic diagram of absorption and reflection of the ZHA/perovskite. **b** Light reflectivity curves of the ZnO/CsPbBr_3_ and the ZHA/CsPbBr_3_. Light absorbance of **c** ZHA/CsPbBr_3_ and **d** ZnO/CsPbBr_3_ with variable incident angles of the light

Figure 2a shows the light transmission process in the ZHA/CsPbBr_3_ thin film. As we known, when light is incident from one medium to another, the light was reflected at the interfaces of layers which reduces the light utilization efficiency. Therefore, the traditional ZnO film, without light trapping structure, could loss a large amount of light due to the reflection (Fig. S2a). However, our ZHA layer with the hollow hemisphere structure can enable the reflection light to be refracted multiple times in the microcavity inside the film (Fig. 2a), which greatly reduces the light reflection (Fig. 2b), enhances the light absorption (Fig. S2b) and further effectively improves the light capture efficiency. From the absorption spectrum, the absorption edge of the perovskite was located at 530 nm, showing the band gap of 2.28 eV (Fig. S3). The relative transmittance (*T*_R_) of the ZnO and the ZHA can be calculated by the relative transmittance formula (Fig. S4), and the value of the *T*_R_ was 26.85 and 4.26 for the ZHA and the ZnO, respectively. Therefore, these results further proved that the single-layer hollow ZnO hemisphere array structure was beneficial for the utilization of light due to the light trapping effect.

More importantly, our ZHA-based structure can enable the absorption of the perovskite behaving broader horizon compared with the traditional ZnO film-based structure, as shown in Fig. 2c, d and S5. With increasing the angle of the incident light, the absorption of planar ZnO/CsPbBr_3_ structure decreases sharply, while that of the ZHA-based device shows a negligible reduction, which is obviously attributed to the light trapping effect of the ZHA structure. Besides, when the incident angle of the light increases, the light absorption of planar devices will gradually decrease until it is all reflected. However, for the ZHA-based device, even if the incident angle of the light reaching 75°, the light absorption still can be up to 80% of the maximum value, showing that our ZHA-based perovskite PDs with superior light trapping capability and will benefit to the broad horizon.

**Fig. 3:**
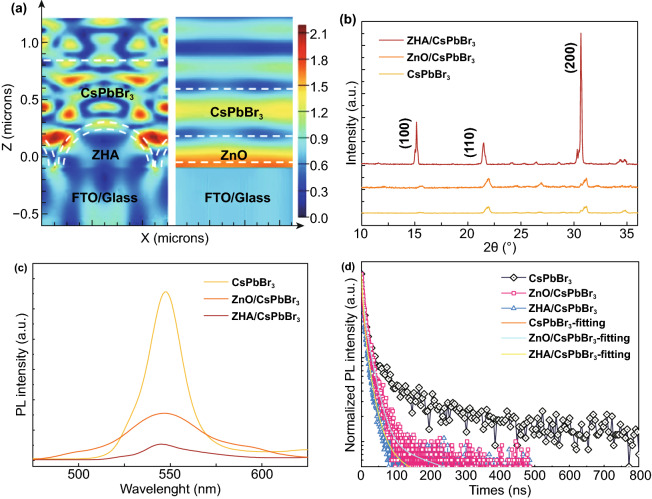
**a** FDTD Simulation of ZHA (left) and planar (right) devices under 550 nm illumination and **b** X-ray diffraction pattern, **c** steady PL, and **d** transient PL based on devices with different structures

The 3D FDTD simulation results of the light absorption distribution are shown in Fig. 3a and S6, S7, which displays that the light absorption of the planar device exhibits a laminar distribution because of the formation of standing wave [45]. The strongest light absorption (dark red part) is located at the ZnO/FTO interface, which means great waste of light utilization. Interestingly, for the ZHA/perovskite device, the light field is mainly distributed in the perovskite active area, indicating that the distribution of the photogenerated carrier is transferred from the ZnO/FTO interface to the perovskite active layer, which benefits for the generation, transport and separation of carriers, improving the light utilization efficiency. Besides, the XRD patterns (Fig. 3b) show that the ZHA-based CsPbBr_3_ film behaves relatively higher intensity on the (100), (110) and (200) planes than that of the other two samples, indicating the ZnO hemisphere array structure could improve the crystallinity quality of the CsPbBr_3_ film due the big space between the hemispheres. This good crystallinity could result in much CsPb_2_Br_5_ precursor solution confined in the space and the high-quality CsPbBr_3_ film was formed [32]. Besides, from the peak intensity of the (100) plane, the grain size of the CsPbBr_3_ prepared on the ZHAs calculated according to the Scherrer formula *D* = *Kλ*/*β* cos*θ* (where *D* is the grain diameter along the direction perpendicular to the crystal plane, *K* is Scherrer constant, *λ* is the wavelength of incident X-ray, *θ* is Bragg diffraction angle, and *β* is the half-peak width of diffraction peak) is as large as 93.89 nm, which is almost 3 times of that of the ZnO/CsPbBr_3_ (33.5 nm) and the bare CsPbBr_3_ film (30.2 nm). The photoluminescence (PL) characterizations of the CsPbBr_3_, the ZHA/CsPbBr_3_ and the ZnO/CsPbBr_3_ samples in Fig. 3c show that the relative intensity of the PL peak of the ZHA/CsPbBr_3_ and ZnO/CsPbBr_3_ samples is much lower than that of the bare CsPbBr_3_ film, and the ZHA/CsPbBr_3_ sample shows the smallest PL intensity, indicating efficient charge transfer at the ZHA/CsPbBr_3_ interface due to the unique structure of the ZHA. Moreover, we observed a small redshift of the PL peak for the CsPbBr_3_ prepared on the ZHAs, which is attributed to the larger grain size [46, 54]. The time-resolved PL (TRPL) decay measurement with three structures is performed and shown in Fig. 3d. All of the TRPL results follow the 3-exponential function, and the values of the parameters are listed in Table S1. Generally, the average decay time (*τ*_ave_) reflects the carrier recombination dynamics in the perovskite layer. For the CsPbBr_3_ film, the ZnO/CsPbBr_3_ and the ZHA/CsPbBr_3_, the *τ*_ave_ of 283, 62 and 14 ns were calculated, respectively, and those values were related to the efficiency of charge extraction by electron transport layer [47]. The extremely short quenching time of ZHA/CsPbBr_3_ devices further proves the advantages of this structure.

**Fig. 4:**
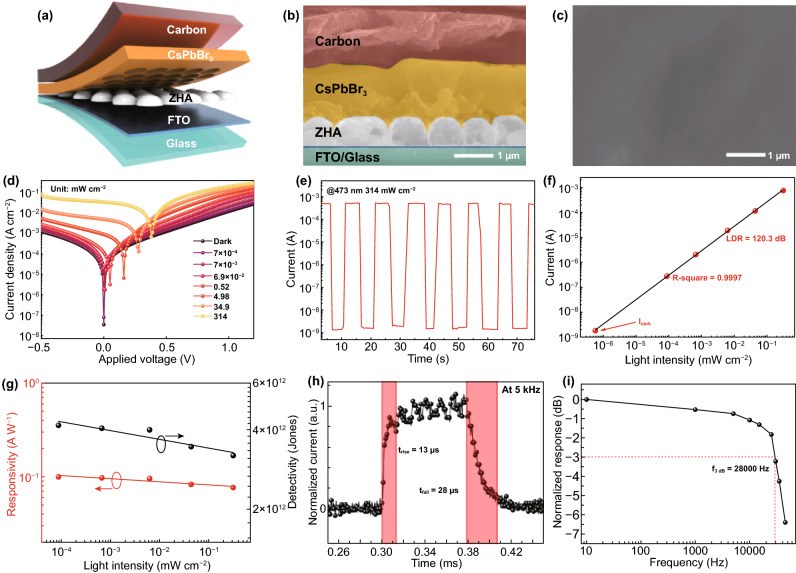
**a** Structure diagram of the ZHA/CsPbBr_3_ PD. **b** Cross-sectional SEM image of the PD. **c** Top-view SEM image of the CsPbBr_3_ layer prepared on the ZHAs. **d** *J–V* curves under different light intensities. **e** *I–t* curve under the light intensity of 314 mW cm^−2^. **f** LDR curve. **g** Responsivity and detectivity. **h** Response time and **i** *f*_−3db_ of the ZHA/CsPbBr_3_ PDs

Because of the merits of the single-layer ZnO hollow hemispheres including reflection reduction, enhancing the crystal quality of the perovskite, the transfer of optical field distribution to active layer, a self-powered perovskite PD based on the single-layer ZnO hollow hemispheres was fabricated and the structure diagram of the device with Glass/FTO/ZHA/CsPbBr_3_/carbon structure is shown in Fig. 4a. The cross-sectional SEM image of the device and the top-view SEM image of CsPbBr_3_ layer prepared on the ZHAs are shown in Fig. 4b, c, respectively. Many large CsPbBr_3_ perovskite grains are formed in the gaps among the hemispheres and a largest perovskite grain is on top, leading to a smooth surface of the perovskite (Fig. S8), which benefits for the device performance and the stability.

Figure 4d shows the current density–voltage (*J*–*V*) curves of the device under the irradiation of various light intensities. From the graph, the open-circuit voltage of the device increases with increasing light intensity, and the device exhibits extremely low dark current density of 3.4 × 10^–8^ A cm^−2^ at zero bias, which can be attributed to the structure of ZnO hollow sphere arrays. Under the illumination of 314 mW cm^−2^, the photocurrent density reaches 2.5 × 10^–2^ A cm^−2^. The extremely low dark current density and high photocurrent density lead to the switching ratio (*J*_ph_/*J*_d_) of the device reaching as high as 1.25 × 10^6^. The current–time (*I-t*) curve of the device is shown in Fig. 4e, which shows that the dark current of the device does not decay during repeated testing, indicating the high stability of the device.

The LDR means that the photodetector behaves the linear response capability under a range of the light intensity, and beyond this range, the intensity of optical signals cannot be accurately detected and calculated. Generally, the LDR can be expressed by the following formula [48]:

1 $$ {\text{LDR}} = 20\log \frac{{I_{{{\text{ph}}}} }}{{I_{d} }} $$

in which *I*_ph_ is the light current and I_d_ is the dark current of the device. By fitting the current values of the device under different light intensities, we get the linear dynamic graph of the device, as shown in Fig. 4f. The *R*^2^ of the device is 0.9997 and the LDR reaches 120.3 dB, these high values show our devices behave good linearity, and the response of the device can be changed linearly in a large range. Responsivity (*R*) and detectivity (D*) are the sensitivity of the PD, and big values of these parameters are very important for the device to detect light signals, especially the weak light. The formula is as follows [49]:

2 $$ R = \frac{{I_{{{\text{ph}}}} }}{{{\text{SL}}_{{{\text{light}}}} }} $$

3 $$ D^{*} = \frac{R}{{\sqrt {2qJ_{d} } }} $$

where *S* is the effective irradiation area of the device and *L*_light_ is the optical power intensity. According to the formula, we get the responsivity and detectivity curves of the device as shown in Fig. 4g. The responsivity of the device shows a biggest value under the light intensity of 7 × 10^–4^ mW cm^−2^, reaching 0.1 A W^−1^, which slightly decreases when increasing the light intensity. Besides, the detectivity of the device shows the biggest value of 4.2 × 10^12^ Jones, which is comparable to or even better than other perovskite PDs [48–52], and the detailed comparison is shown in Table 1. In addition, our PDs show fast response speed with the rise/falling time of 13/28 µs (Fig. 4h), and these small values are attributed to the structure of the single-layer hollow ZnO hemisphere arrays, which enables the main distribution of the optical field in the perovskite and greatly promotes the migration of carriers, especially photogenerated holes. Furthermore, the device response with various illumination frequencies (10 Hz–50 kHz) was performed as shown in Fig. S10, and the *f*_−3dB_ of the device is calculated and the value reaches 28 kH, this big value further confirms the fast response speed of our PDs [53].

**Table 1:** Comparison of the perovskite PD performance

Device structure	Switching ratio	Responsivity (A W^−1^)	Detectivity (Jones)	Rise/fall time (ms)	References
FTO/SnO_2_/MAPbI_3_/carbon	2 × 10^5^	0.26	7.01 × 10^11^	0.03/0.3	[48]
FTO/MgO/ZnO/MAPbI_3_/carbon	7.0 × 10^4^	0.06	1.5 × 10^12^	0.63/1.6	[50]
FTO/SnO_2_/CsPbBr_3_/carbon	1.54 × 10^6^	0.11	1.4 × 10^12^	0.006/0.064	[23]
Au/CuI/CsPbBr_3_/Au	1.5 × 10^3^	0.28	6.2 × 10^10^	0.04/2.96	[51]
Au/CsPbBr_3_-CsPbI_3_/Au	1 × 10^5^	20	-	0.7/0.8	[52]
FTO/ZnO/CsPbBr_3_/carbon	3.93 × 10^4^	0.08	3.14 × 10^11^	0.12/0.045	This work
FTO/ZHA/CsPbBr_3_/carbon	1.04 × 10^6^	0.1	4.2 × 10^12^	0.013/0.028	This work

**Fig. 5:**
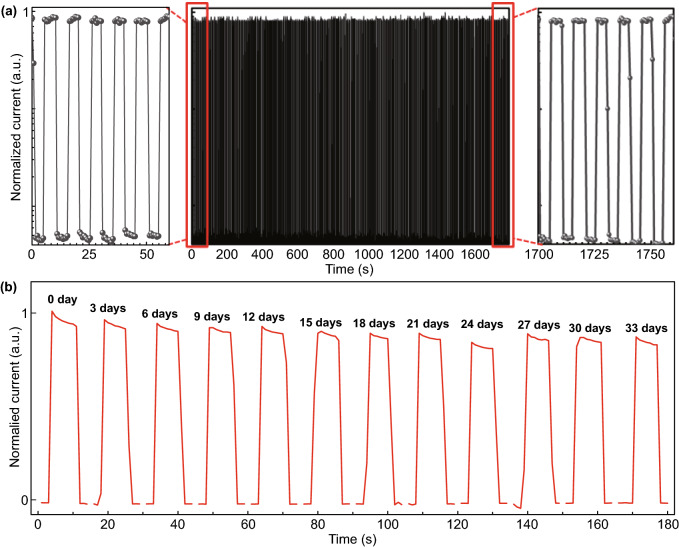
**a** Longtime irradiation test and **b** storage stability of the unencapsulated device for 33 days

The stability of the PDs is very important for the industrial application, and we measured the longtime illumination stability of the ZHA-CsPbBr_3_ device under the bias voltage of 0.01 V, as shown in Fig. 5a. Under the light intensity of 440 mW cm^−2^ for 1800s, the photocurrent and dark current show no change in the first 60 s (left in Fig. 5a) and the last 60 s (right in Fig. 5a), which displays the long-term stability of the device under illumination. Besides, the storage stability of the unencapsulated device in air for a long time is shown in Fig. 5b. The device was placed in the air with an average humidity of 70% and the temperature of 32 °C for 33 days. As shown in Fig. 5b, the performance of our unencapsulated PDs only decreased by about 10% of its initial efficiency within 30 days, showing ultrahigh stability of our devices.

**Fig. 6:**
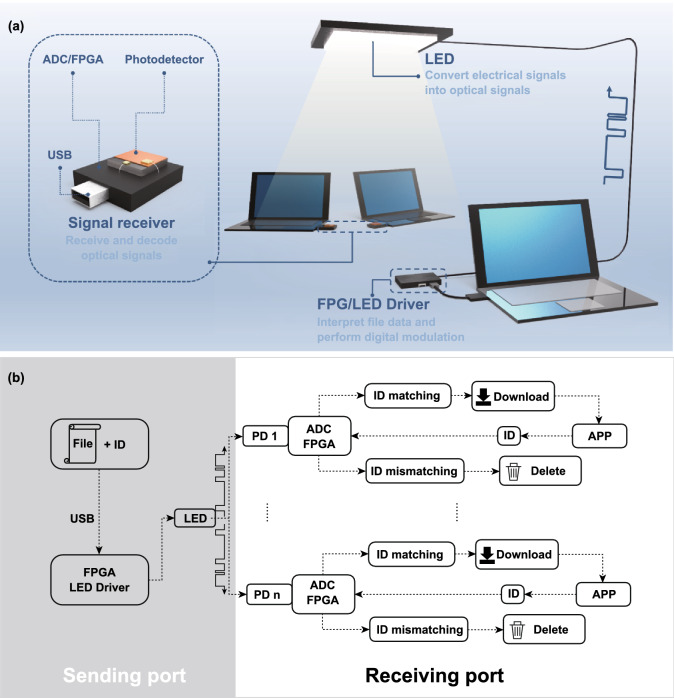
**a** Schematic and **b** mechanism diagram of transmitting confidential documents by optical communication

The high *f*_−3db_ response and wide-angle detection range encourage us to fabricate ZHA-CsPbBr_3_ PDs to optical communication. The optical communication technology, which can quickly construct anti-interference and anti-interception security information space, shows great attraction for replacing the wireless fidelity in highly secure areas [23, 54]. According to some important confidential documents, they are not allowed to be transmitted through the network or USB flash drive. The use of optical communication can well solve the problem of file transmission security. The schematic diagram of transmitting confidential documents by optical communication is shown in Fig. 6a, and the mechanism diagram of the file transmission is shown in Fig. 6b. At the file sending port, a desktop application program written by C++ based on Qt framework is used, and the files to be sent and the ID number of the receiver are selected by the desktop application program (the application operation page is shown in Fig. S11). Moreover, the file and ID number are packaged and sent to the cache of the FPGA master controller, which are connected with the computer through USB. Then the FPGA master controller reads out the file data for digital modulation; meanwhile, the modulation signal is sent out in the form of optical signal by controlling the high-speed flashing of the LED. At the receiving port, the PD receives optical signals and converts them into electrical signals. The analog electrical signals are converted into digital signals through the analog-to-digital converter (ADC) and sent to the FPGA slave controller. Then the signals are demodulated by the FPGA slave controller, and if the ID information matches with the host ID, the files will be accepted and transmitted to the computer through USB. On the contrary, the FPGA clears the received file data from the controller and ensures the precise transmission of the file. Finally, we constructed an optical communication system with an average transmission rate of 10 Kb s^−1^. During the actual operation, the received and sent files were exactly the same, and the zero packet loss rate was achieved. The detailed information can be seen from Fig. S11.

## Conclusions

In summary, we introduced single-layer hollow ZHAs fabricated by using a PS microsphere template, which reduce the reflection, enhance the incident angle of light and especially transfer the distribution of the optical field. Finally, efficient devices with enhanced performances were obtained. Our optimized PDs showed high self-powered performance with a LDR of 120.3 dB, a detectivity of 4.2 × 10^12^ Jones, rise/fall time of 13/28 µs and the *f*_−3 dB_ of up to 28 kHz. Furthermore, the PD was applied to the directional transmission of encrypted files as the signal receiving port with super high accuracy. This work uniquely combines the features of high-performance self-powered perovskite PDs with optical communication technology, paving the path to wide applications of all-inorganic perovskite PDs.

## Supplementary Information

Below is the link to the electronic supplementary material.

Supplementary material 1 (DOCX 1391 kb)
