# Supplementary material for: Single-Layer ZnO Hollow Hemispheres Enable High-Performance Self-Powered Perovskite Photodetector for Optical Communication
Source: Nanomicro Lett. 2021 Feb 12;13:70. doi: 10.1007/s40820-021-00596-5 (PMC8187591; doi:10.1007/s40820-021-00596-5)
Supplement: Supplementary file 1 — Supplementary material 1 (DOCX 1391 kb) [file 40820_2021_596_MOESM1_ESM.pdf]

Supporting Information for

Single-Layer ZnO Hollow Hemispheres Enable High-Performance Self-Powered Perovskite Photodetector for Optical Communication

Xiyan Pan[1,#] Jianqiang Zhang[1,#] Hai Zhou[1,*], Ronghuan Liu[1], Dingjun Wu[1], Rui Wang[1], Liangping Shen[1], Li Tao[1], Jun Zhang[1], Hao Wang[1,*]

[1]Hubei Key Laboratory of Ferro & Piezoelectric Materials and Devices, School of Microelectronics and Faculty of Physics and Electronic Science, Hubei University, Wuhan 430062, P. R. China

[#]Xiyan Pan and Jianqiang Zhang contributed equally to this work

*Corresponding authors. E-mail: hizhou@hubu.edu.cn (Hai Zhou), wangh@hubu.edu.cn (Hao Wang)

Supplementary Tables and Figures

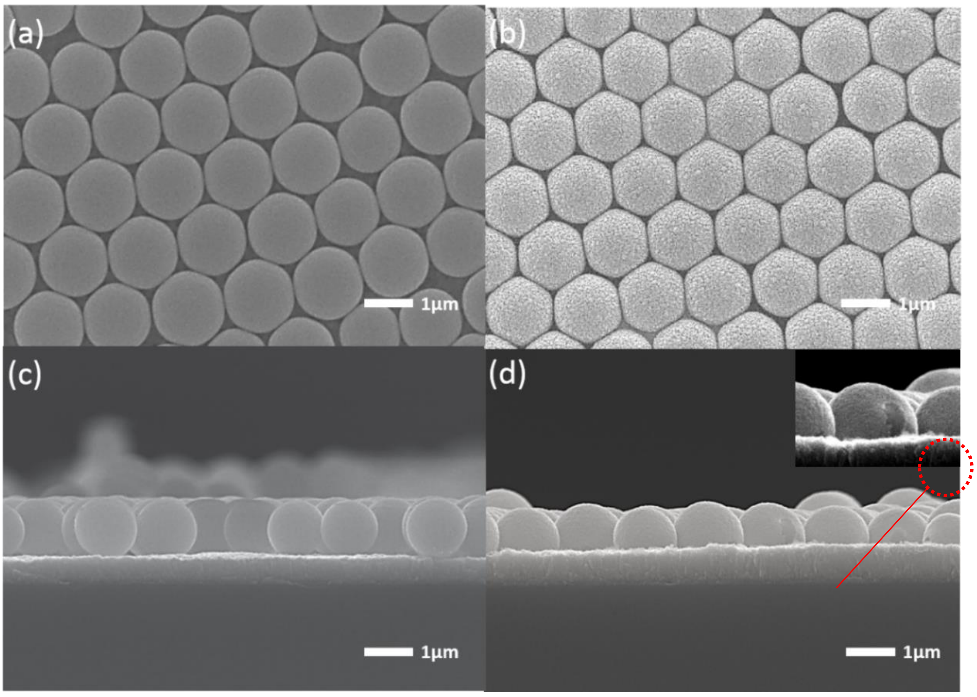

Fig. S1 Top-view (**a**) and cross-sectional (**c**) SEM images of the single-layer PS balls on FTO substrate. Top-view (**b**) and cross-sectional (**d**) SEM images of ZHA layer after annealing (the insert figure of (**d**) shows that the ZnO hemispheres are hollow)

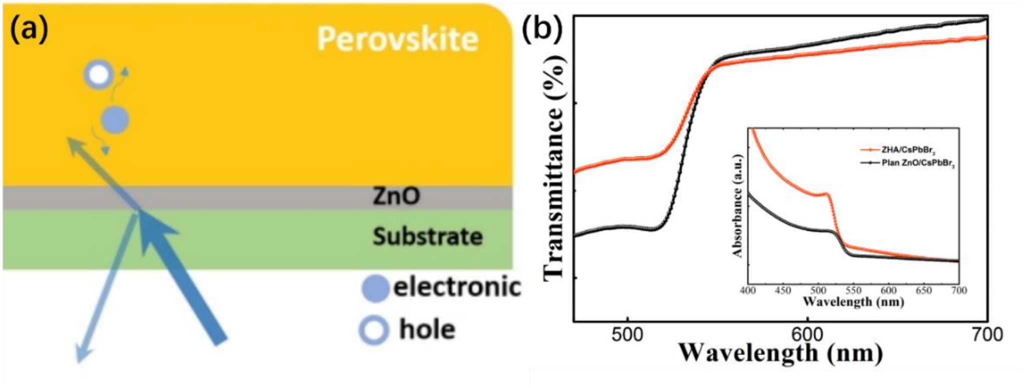

Fig. S2 (**a**) Schematic diagram of absorption light of planar ZnO film. (**b**) Light absorption (the insert of the figure) and transmittance curves of the ZnO/$CsPbBr_3$ and the ZHA/$CsPbBr_3$

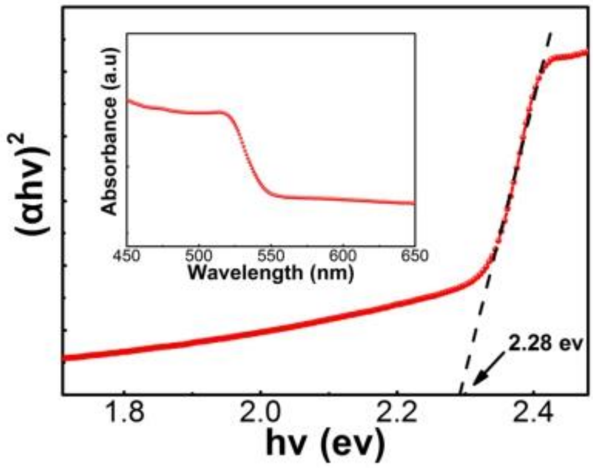

Fig. S3 Optical absorbance and band gap of the ZHA-$CsPbBr_3$ film

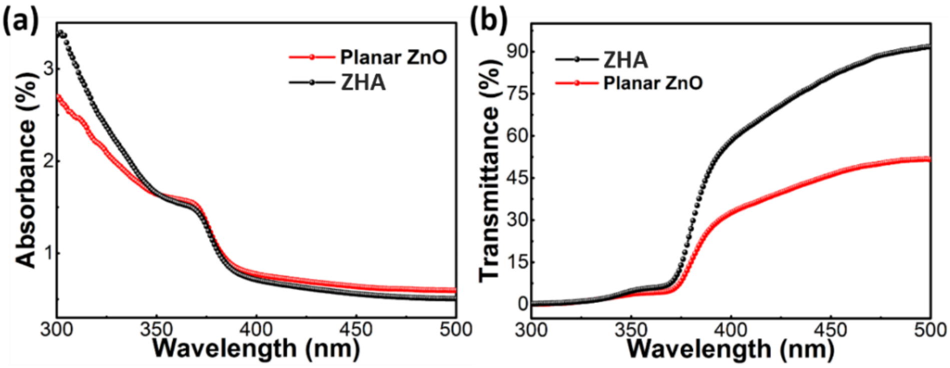

Fig. S4 (**a**) Absorbance and (**b**) transmittance of the ZHA layer and the planar ZnO layer

Discussion: The ZnO electrode modified layer shows certain interface light reflection, which will affect the absorption and the utilization of incident light for the perovskite films. Therefore, the preparation of ZnO electrode modified layer with

wide spectrum and high transmittance can effectively improve the performance of perovskite devices. Liang [S1] calculated the relative light transmittance T_R of the ZnO electrode modified layer to study the performance of the devices. The relative light transmittance calculation formula is as follows:

$$T_R = \frac{\int_{\lambda_2}^{\lambda_1} I_\lambda t_\lambda \, d\lambda}{\int_{\lambda_2}^{\lambda_1} I_\lambda \, d\lambda} \qquad \text{(S1)}$$

In the formula, t_λ refers to the transmittance of FTO/ZnO substrate at λ, I_λ refers to the absorbance of FTO/ZnO substrate at λ, and λ_1-λ_2 is the measured wavelength range. T_R can well reflect the utilization of light through the modified electrode layer. The larger T_R means that more light is absorbed by the active layer, which can improve the performance of the devices. We measured the absorption and transmission of the ZHA and the planar ZnO. As shown in Figure S4, under the almost equal condition, the transmittance of the ZHA is much higher than that of planar ZnO, which is due to the hemisphere array structure and more light signals will reach the perovskite layer, benefiting the device performance. Substituting the test data into the formula, the T_R of ZHA layer and the planar ZnO layer are calculated as T_R (ZHA)=26.85, T_R (ZnO)=14.26, respectively.

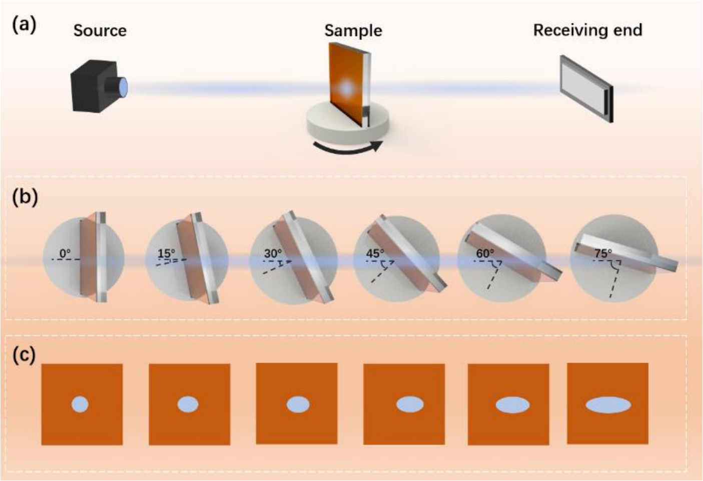

Fig. S5 (**a**) Schematic diagram of the light absorption test with variable angles. The variable angles (**b**) and the light-receiving area (**c**) of the device

Figure S5 shows the schematic diagram of light absorption test. With the rotation of the device, the incident light angle changes, and the light receiving area of the device increases with the increase of the incident light angle. As shown in Figure S5c, the relative absorption value of the device can be obtained by the following formula:

$$Abs = \frac{S_0 \cdot Abs^*}{S} = Abs^* \cdot \cos\theta \qquad \text{(S2)}$$

where Abs is the relative absorbance, Abs^* is the measured absorbance value, S_0 is the illumination area when the deflection angle is zero, θ is the incident angle, and S is illumination area when deflection angle is θ.

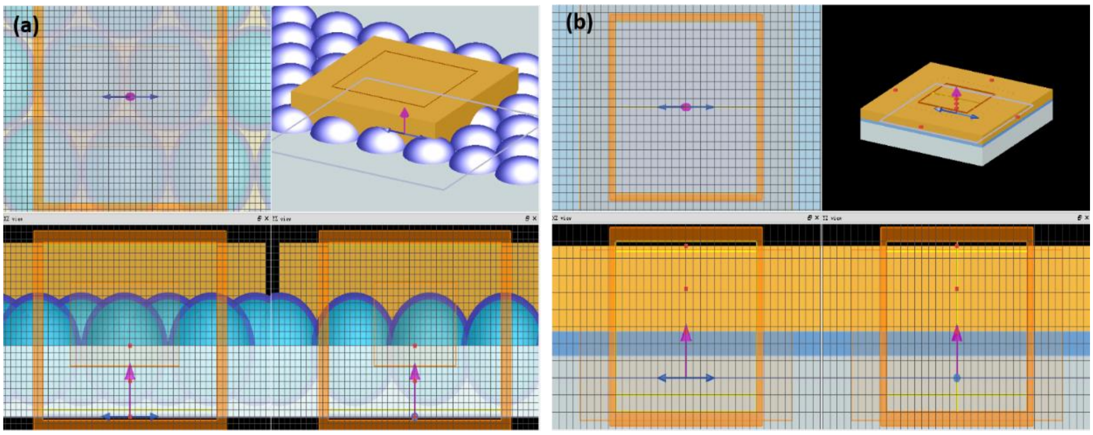

Fig. S6 Simulation model graphics of (**a**) the ZHA and (**b**) planar ZnO

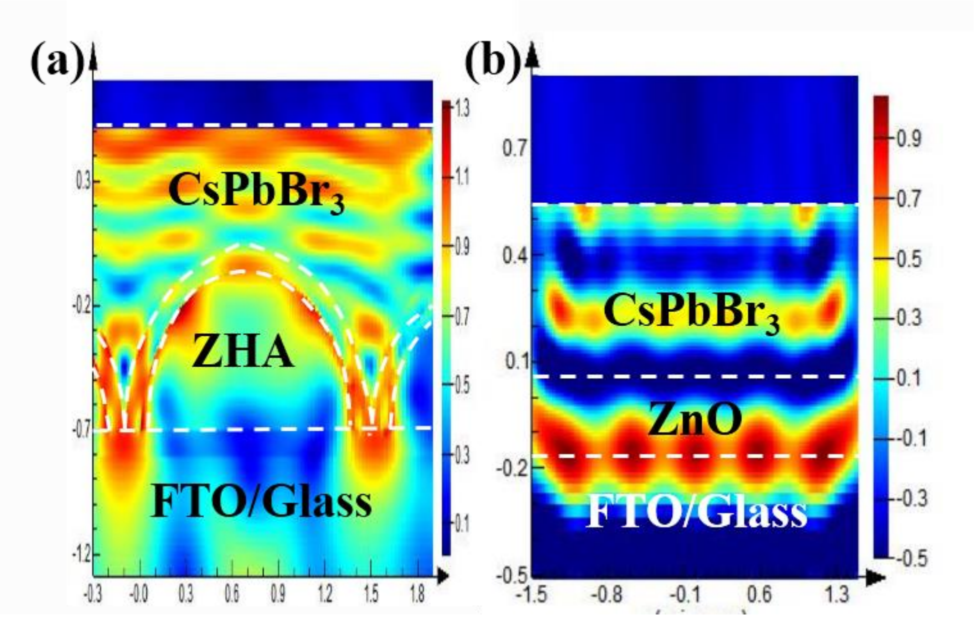

Fig. S7 FDTD Simulation of ZHA (**a**) and planar (**b**) devices under 473 nm illumination

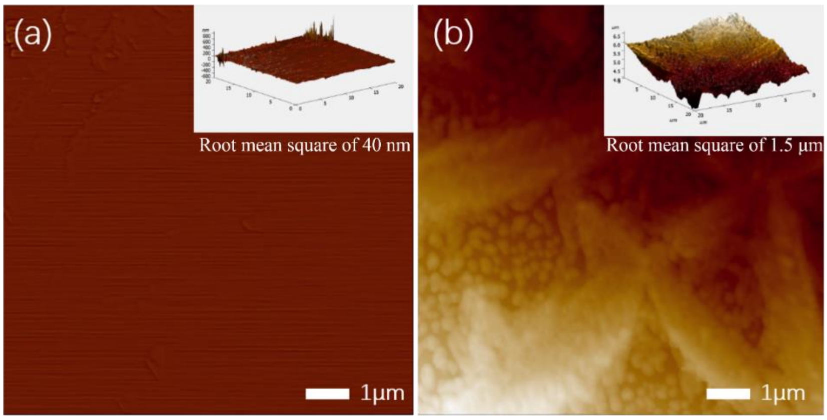

Fig. S8 Surface topography of the $CsPbBr_3$ on (**a**) the ZHA and (**b**) the planar ZnO measured by atomic force microscope

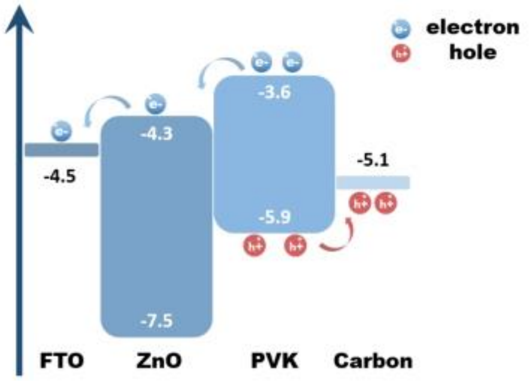

Fig. S9 Energy band structure of our devices

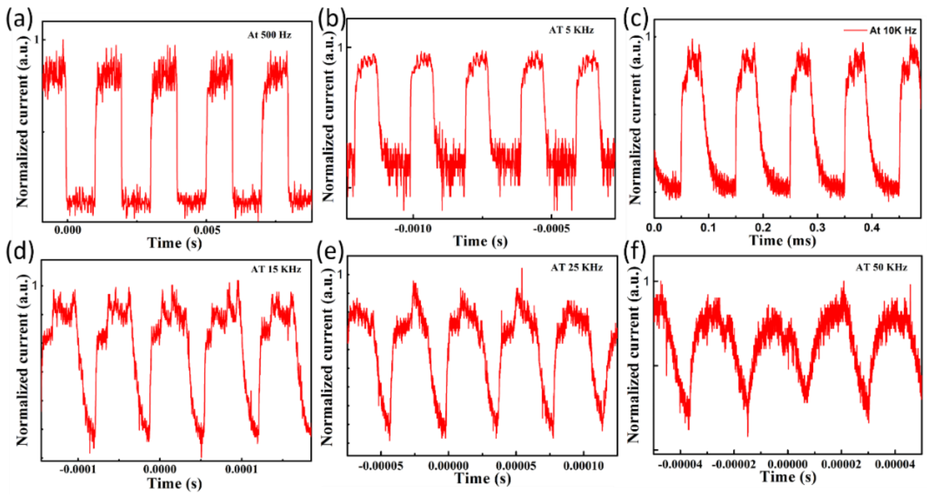

Fig. S10 *I-t* curves under variable frequency lighting

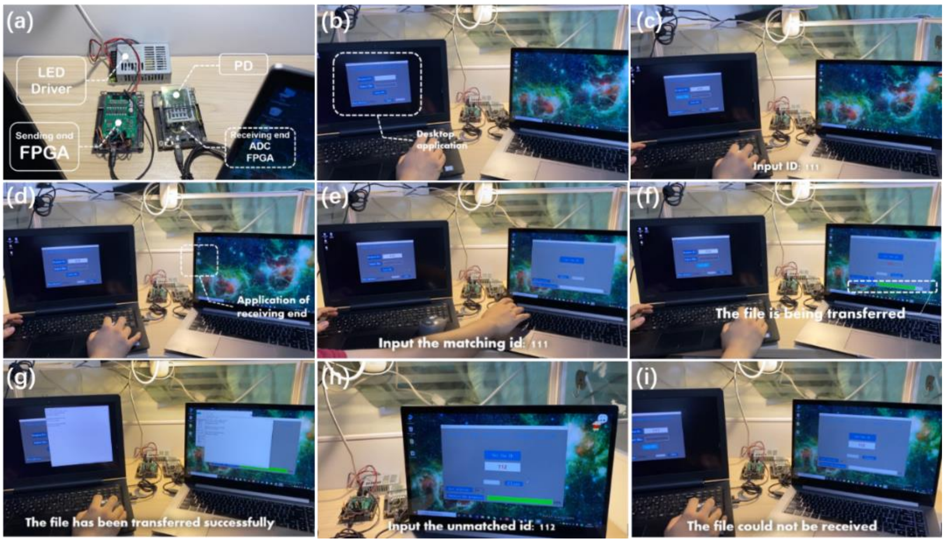

Fig. S11 Photographs of the optical communication system demonstration

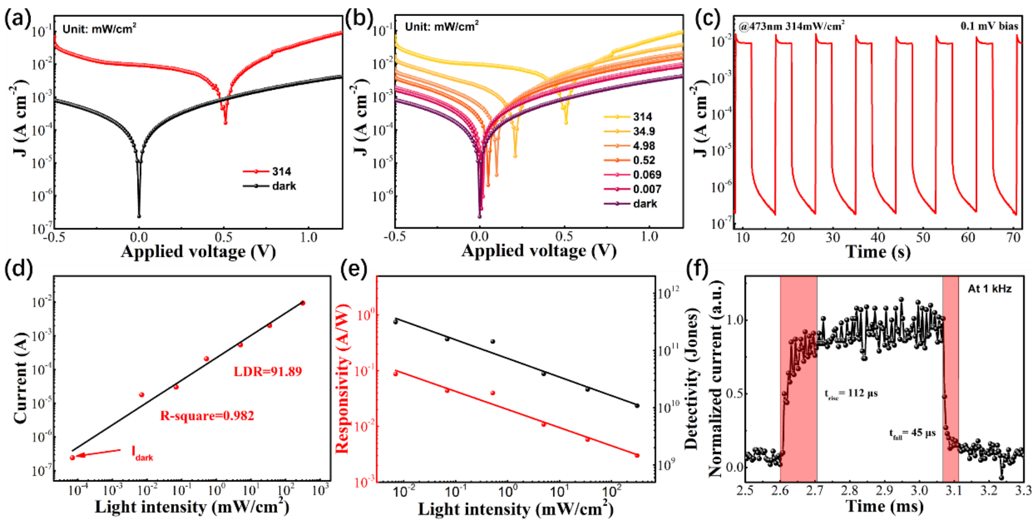

Fig. S12 Device performance of the planar-ZnO/$CsPbBr_3$. (**a**) J-V curves under light intensity of 314 mW cm^{-2}. (**b**) *J-V* curves under different light intensities. (**c**) I-t curve under light intensity of 314 mW cm^{-2} at 0.1 mV. (**d**) LDR curve. (**e**) Responsivity and detectivity. (**f**) Response time

Table S1 Photoluminescent carrier lifetime of different samples

Sample	τ_1 (ns)/A_1	τ_2 (ns)/A_2	τ_3 (ns)/A_3	τ_{ave} (ns)
ZHA-$CsPbBr_3$	3.97/0.54	12.96/0.41	39.91/0.04	14.59
ZnO-$CsPbBr_3$	4.39/0.55	19.22/0.43	247.38/0.02	62.24
Bare $CsPbBr_3$	1.52/0.63	19.36/0.34	421.43/0.03	283.91

PL Decay

After fitting the PL decay curves of different films, the average carrier life is calculated by the formula [S2]

$$\tau_{ave} = \frac{A_1\tau_1{}^2+A_2\tau_2{}^2+A_3\tau_3{}^2}{A_1\tau_1+A_2\tau_2+A_3\tau_3} \quad \text{(S3)}$$

where *A*1, *A*2, and *A*3 are relative amplitudes of carrier lifetime, τ_1 and τ_2 represent the fast decay component, which shows the bulk recombination in perovskite crystal, while τ_3 represents the slow decay component, which shows the recombination of free carriers in radiation channel [S3-S5].

Supplementary References

[S1] Z. Liang, Q. Zhang, O. Wiranwetchayan, J. Xi, Z. Yang et al., Effects of the morphology of a ZnO buffer layer on the photovoltaic performance of inverted polymer solar cells. Adv. Funct. Mater. **22**(10), 2194-2201 (2012). https://doi.org/10.1002/adfm.201101915

[S2] D. Wu, H. Zhou, Z. Song, M. Zheng, R. Liu et al., Welding perovskite nanowires for stable, sensitive, flexible photodetectors. ACS Nano **14**(3), 2777-2787

(2020). https://doi.org/10.1021/acsnano.9b09315

[S3] D. Shi, V. Adinolfi, R. Comin, M. Yuan, E. Alarousu et al., Low trap-state density and long carrier diffusion in organolead trihalide perovskite single crystals. Science **347**(6221), 519-522 (2015). https://doi.org/10.1126/science.aaa2725

[S4] S. Zhou, R. Tang, L. Yin, Slow-photon-effect-induced photoelectrical-conversion efficiency enhancement for carbon-quantum-dot-sensitized inorganic $CsPbBr_3$ inverse opal perovskite solar cells. Adv. Mater. **29**(43), 1703682 (2017). https://doi.org/10.1002/adma.201703682

[S5] H. Cho, S.-H. Jeong, M.-H. Park, Y.-H. Kim et al., Wolf C overcoming the electroluminescence efficiency limitations of perovskite light-emitting diodes. Science **350**(6265), 1222-1225 (2015). https://doi.org/10.1126/science.aad1818
